# Crystallographic and spectroscopic characterization of racemic Mosher’s Acid

**DOI:** 10.1107/S2056989020008403

**Published:** 2020-06-26

**Authors:** Carolyn Z. Savich, Joseph M. Tanski

**Affiliations:** a Department of Chemistry, Vassar College, Poughkeepsie, NY 12604, USA

**Keywords:** crystal structure, hydrogen bonding, benzoic acid derivatives, tri­fluoro­methyl group

## Abstract

The title compound, C_10_H_9_F_3_O_3_, represents the structure of racemic Mosher’s Acid (systematic name: 3,3,3-tri­fluoro-2-meth­oxy-2-phenyl­propanoic acid), a carb­oxy­lic acid that when resolved can be employed as a chiral derivatizing agent. The compound contains a carb­oxy­lic acid group, a meth­oxy group and a tri­fluoro­methyl substituent on an asymmetric benzylic carbon atom. The two independent mol­ecules in the asymmetric unit form a non-centrosymmetric homochiral dimer *via* inter­molecularly hydrogen-bonded head-to-tail dimers with graph-set notation *R*_2_^2^(8) and donor–acceptor hydrogen-bonding distances of 2.6616 (13) and 2.6801 (13) Å.

## Abstract 2

Mosher’s Acid (systematic name: 3,3,3-tri­fluoro-2-meth­oxy-2-phenyl­propanoic acid) is a carb­oxy­lic acid that when resolved can be employed as a chiral derivatizing agent. The two independent mol­ecules in the asymmetric unit form a non-centrosymmetric homochiral dimer *via* inter­molecularly hydrogen-bonded head-to-tail dimers with graph-set notation 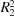
(8).

## Chemical context   

The title compound, α-meth­oxy-α-tri­fluoro­methyl­phenyl­acetic acid, or 3,3,3-tri­fluoro-2-meth­oxy-2-phenyl­propanoic acid, MTPA (I) is commonly known as Mosher’s Acid. Mosher’s Acid is an aromatic compound in which an asymmetric benzylic carbon atom is specifically substituted with a carb­oxy­lic acid group, a meth­oxy group and a tri­fluoro­methyl substituent. When resolved and in its acid chloride form, it has been shown to be useful as a chiral derivatizing agent (CDA) with natural organic compounds (Cimmino *et al.*, 2017 ▸). Originally, Mosher’s Acid chloride was used to convert a mixture of enanti­omers of amines or alcohols into diastereomeric amides or esters, respectively, in order to analyze the qu­anti­ties of each enanti­omer present within the sample by NMR (Dale *et al.*, 1969 ▸), and also to elucidate the absolute stereochemistry of the starting material (Allen *et al.*, 2008 ▸). Mosher’s Acid has recently been used in NMR studies of the ring flip in the atrane cages of Group 14 metallatranes, where as an axial substituent it forces the Δ- and Λ-isomers to become diastereomeric (Glowacki *et al.*, 2019 ▸). The synthesis of Mosher’s Acid reported in early work converted phenyl tri­fluoro­methyl ketone to α-tri­fluoro­methyl­phenyl­aceto­nitrile with sodium cyanide and methyl sulfate followed by treatment with concentrated sulfuric acid to obtain the acid (Dale *et al.*, 1969 ▸). More recently, Mosher’s Acid was obtained by treatment of phenyl tri­fluoro­methyl ketone with tri­methyl­silyl tri­chloro­acetate followed by hydrolysis (Goldberg & Alper, 1992 ▸).
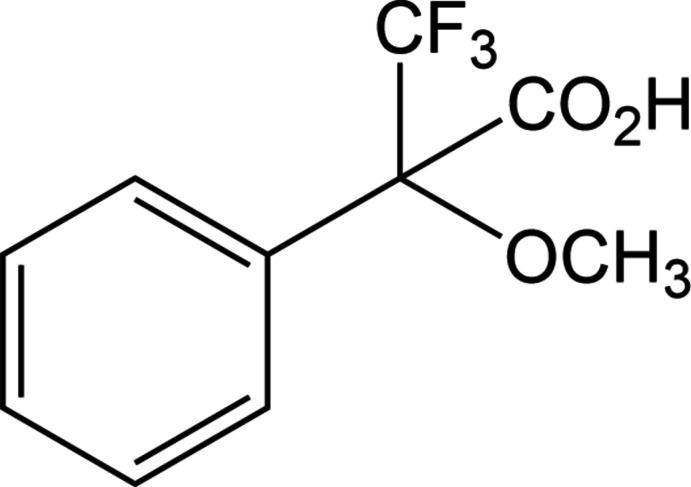


## Structural commentary   

**Figure 1:**
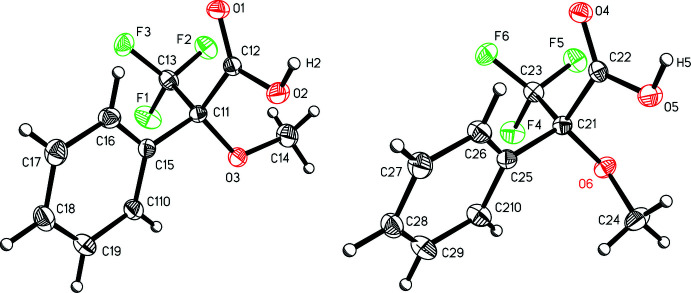
A view of the two independent mol­ecules of 3,3,3-tri­fluoro-2-meth­oxy-2-phenyl­propanoic acid (I), oriented so as to highlight the different conformations of the meth­oxy group. Displacement ellipsoids are shown at the 50% probability level.

The mol­ecular structure of the title compound (Fig. 1 ▸) reveals that there are two independent mol­ecules in the asymmetric unit. Each consists of a mono-substituted benzene ring including a meth­oxy group, a tri­fluoro­methyl group, and a carb­oxy­lic acid on the asymmetric benzylic carbon atom. The mol­ecules show slightly different conformations, specifically in regard to the disposition of the meth­oxy group. In the mol­ecule with asymmetric carbon C11, the meth­oxy group is canted away from the phenyl ring, with a C15—C11—O3—C14 torsional angle of −175.55 (12)°. In the other mol­ecule, the meth­oxy group is bent in, with a C25—C21—O6—C24 torsional angle of −51.12 (15)°.

## Supra­molecular features   

**Figure 2:**
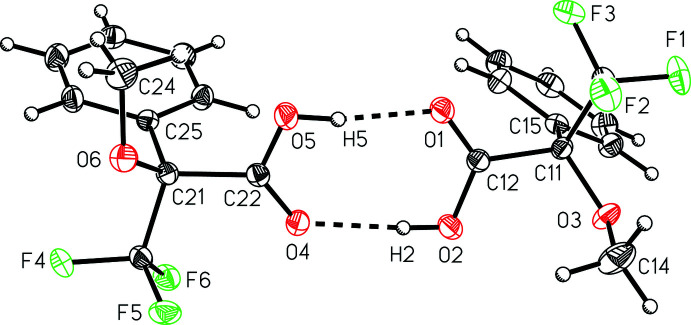
A view of the inter­molecular hydrogen bonding in 3,3,3-tri­fluoro-2-meth­oxy-2-phenyl­propanoic acid (I).

**Figure 3:**
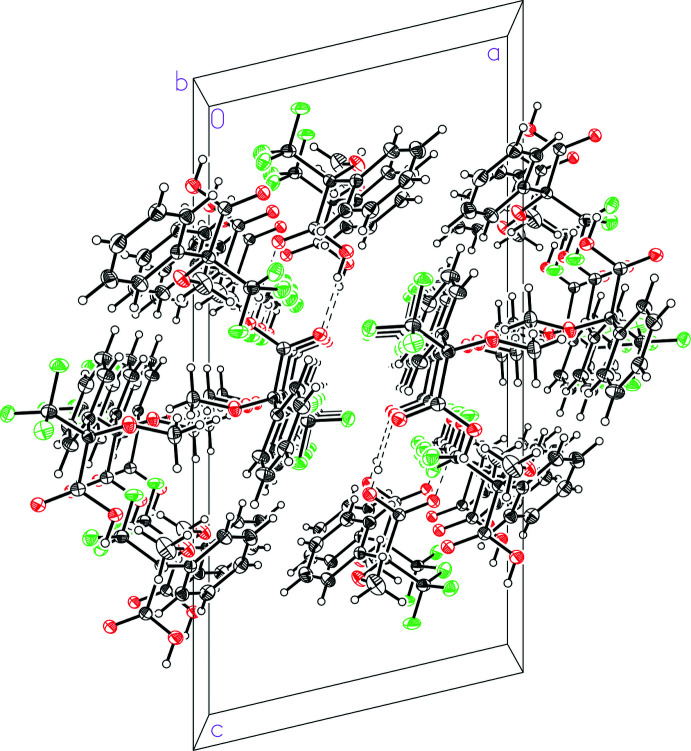
A view of the packing in 3,3,3-tri­fluoro-2-meth­oxy-2-phenyl­propanoic acid (I).

Although the material is racemic, two independent mol­ecules of the same chirality are observed to hydrogen bond together into pairwise dimers (Table 1 ▸, Fig. 2 ▸), with graph-set notation 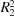
(8) and donor–acceptor hydrogen-bonding distances of 2.6616 (13) and 2.6801 (13) Å. The dimers further pack together *via* van der Waals inter­actions without any other notable inter­molecular inter­actions such as π-stacking or fluorine–fluorine contacts less than the sum of the van der Waals radii. The hydrogen-bonded dimers stack along the crystallographic *b*-axis direction (Fig. 3 ▸).

## Database survey   

The Cambridge Structural Database (Version 5.40, update of March 2020; Groom *et al.*, 2016 ▸) contains no structures of racemic or resolved Mosher’s Acid itself. However, there are numerous structures of its carboxyl­ate salts, and one example (UTUHUN) of the neutral acid co-crystallized with an imidazole (Tydlitát *et al.*, 2010 ▸). In this example, the bond lengths about the asymmetric carbon atom are similar to those observed in (I), with C—CO_2_H = 1.547 (5), C—CF_3_ = 1.538 (6), C—C_Ar_ 1.519 (5) and C—OCH_3_ 1.419 (5) Å, while the disposition of the meth­oxy group with a torsional angle of 170.02° is most similar to the unique mol­ecule in (I) with asymmetric carbon atom C11.

## Synthesis and crystallization   

Racemic 3,3,3-tri­fluoro-2-meth­oxy-2-phenyl­propanoic acid (99%) was purchased from Aldrich Chemical Company, USA, and was used as received.

## Refinement   

Crystal data, data collection and structure refinement details are summarized in Table 2 ▸. All non-hydrogen atoms were refined anisotropically. Hydrogen atoms on carbon were included in calculated positions and refined using a riding model with C—H = 0.95 and and 0.98 Å and *U*_iso_(H) = 1.2 and 1.5 × *U*_eq_(C) of the aryl and methyl C atoms, respectively. The positions of the carb­oxy­lic acid hydrogen atoms were found in the difference map and the atom refined semi-freely using a distance restraint *d*(O—H) = 0.84 Å, and *U*_iso_(H) = 1.2 × *U*_eq_(O).

## Analytical data   

^1^H NMR (Bruker Avance III HD 400 MHz, CDCl_3_): δ 3.57 (*s*, 3 H, OC*H*_3_), 7.42–7.46 (*m*, 3 H, C_ar­yl_ *H*), 7.57–7.61 (*m*, 2 H, C_ar­yl_ *H*), 9.8 (*br s*, 1 H, O*H*). ^13^C NMR (^13^C{^1^H}, 100.6 MHz, CDCl_3_): δ 55.56 (*s*, *C*H_3_), 84.38 (*q*, *J*_C–F_ = 28 Hz, *C*), 125.94 (*q*, *J*_C–F_ = 292 Hz, *C*F_3_), 127.39 (*s*, *C*_ar­yl_H), 128.68 (*s*, *C*_ar­yl_H), 130.01 (*s*, *C*_ar­yl_H), 131.08 (*s*, *C*_ar­yl_), 170.90 (*s*, *C*OOH). IR (Thermo Nicolet iS50, ATR, cm^−1^): (3700–2700 *v br*, O—H *str*), 3069 (*m*, C_ar­yl_—H *str*), 2955 (*m*, C_alk­yl_—H *str*), 2852 (*m*), 2642 (*w*), 1733 (*v s*, C=O str), 1499 (*m*), 1453 (*m*), 1408 (*m*), 1271 (*s*), 1170 (*s*), 1124 (*s*), 1082 (*m*), 1013 (*s*), 987 (*m*), 959 (*m*), 919 (*w*), 765 (*m*), 704 (*s*). GC–MS (Agilent Technologies 7890A GC/5975C MS): *M* ^+^ = 248 amu, corresponding to the methyl ester of (I), prepared from the parent carb­oxy­lic acid using a literature procedure (Di Raddo, 1993 ▸).

## Supplementary Material

Crystal structure: contains datablock(s) global, I. DOI: 10.1107/S2056989020008403/pk2638sup1.cif

Structure factors: contains datablock(s) I. DOI: 10.1107/S2056989020008403/pk2638Isup2.hkl

Supporting information file. DOI: 10.1107/S2056989020008403/pk2638Isup3.cml

CCDC reference: 2011722

Additional supporting information:  crystallographic information; 3D view; checkCIF report

## Figures and Tables

**Table 1:** Hydrogen-bond geometry (Å, °)

*D*—H⋯*A*	*D*—H	H⋯*A*	*D*⋯*A*	*D*—H⋯*A*
O2—H2⋯O4	0.83 (1)	1.83 (1)	2.6616 (13)	173 (2)
O5—H5⋯O1	0.87 (1)	1.83 (1)	2.6801 (13)	169 (2)

**Table 2:** Experimental details

Crystal data	
Chemical formula	C_10_H_9_F_3_O_3_
*M*_r_	234.17
Crystal system, space group	Monoclinic, *P*2_1_/*n*
Temperature (K)	125
*a*, *b*, *c* (Å)	10.5916 (6), 9.2081 (5), 20.9930 (12)
β (°)	103.304 (1)
*V* (Å^3^)	1992.47 (19)
*Z*	8
Radiation type	Mo *K*α
μ (mm^−1^)	0.15
Crystal size (mm)	0.20 × 0.10 × 0.04

Data collection	
Diffractometer	Bruker APEXII CCD
Absorption correction	Multi-scan (*SADABS*; Krause *et al.*, 2015 ▸)
*T*_min_, *T*_max_	0.92, 0.99
No. of measured, independent and observed [*I* > 2σ(*I*)] reflections	48631, 6071, 4730
*R*_int_	0.039
(sin θ/λ)_max_ (Å^−1^)	0.715

Refinement	
*R*[*F* ^2^ > 2σ(*F* ^2^)], *wR*(*F* ^2^), *S*	0.040, 0.115, 1.02
No. of reflections	6071
No. of parameters	295
No. of restraints	2
H-atom treatment	H atoms treated by a mixture of independent and constrained refinement
Δρ_max_, Δρ_min_ (e Å^−3^)	0.52, −0.38

Computer programs: *APEX2* and *SAINT* (Bruker, 2017 ▸), *SHELXT2014/5* (Sheldrick, 2015*a* ▸), *SHELXL2016/6* (Sheldrick, 2015*b* ▸), *SHELXTL2014* (Sheldrick, 2008 ▸), *OLEX2* (Dolomanov *et al.*, 2009 ▸), and *Mercury* (Macrae *et al.*, 2020 ▸).

## supplementary crystallographic information

### Crystal data

**Table:**

C_10_H_9_F_3_O_3_	*F*(000) = 960
*M**_r_* = 234.17	*D*_x_ = 1.561 Mg m^−^^3^
Monoclinic, *P*2_1_/*n*	Mo *K*α radiation, λ = 0.71073 Å
*a* = 10.5916 (6) Å	Cell parameters from 9803 reflections
*b* = 9.2081 (5) Å	θ = 2.4–30.5°
*c* = 20.9930 (12) Å	µ = 0.15 mm^−^^1^
β = 103.304 (1)°	*T* = 125 K
*V* = 1992.47 (19) Å^3^	Plate, colourless
*Z* = 8	0.20 × 0.10 × 0.04 mm

### Data collection

**Table:**

Bruker APEXII CCD diffractometer	6071 independent reflections
Radiation source: fine-focus sealed tube	4730 reflections with *I* > 2σ(*I*)
Graphite monochromator	*R*_int_ = 0.039
Detector resolution: 8.3333 pixels mm^-1^	θ_max_ = 30.5°, θ_min_ = 2.0°
φ and ω scans	*h* = −14→15
Absorption correction: multi-scan (*SADABS*; Krause *et al.*, 2015)	*k* = −13→13
*T*_min_ = 0.92, *T*_max_ = 0.99	*l* = −29→29
48631 measured reflections	

### Refinement

**Table:**

Refinement on *F*^2^	Primary atom site location: dual
Least-squares matrix: full	Secondary atom site location: difference Fourier map
*R*[*F*^2^ > 2σ(*F*^2^)] = 0.040	Hydrogen site location: mixed
*wR*(*F*^2^) = 0.115	H atoms treated by a mixture of independent and constrained refinement
*S* = 1.02	*w* = 1/[σ^2^(*F*_o_^2^) + (0.0566*P*)^2^ + 0.9577*P*] where *P* = (*F*_o_^2^ + 2*F*_c_^2^)/3
6071 reflections	(Δ/σ)_max_ = 0.001
295 parameters	Δρ_max_ = 0.52 e Å^−^^3^
2 restraints	Δρ_min_ = −0.38 e Å^−^^3^

### Special details

**Table:**

Geometry. All esds (except the esd in the dihedral angle between two l.s. planes) are estimated using the full covariance matrix. The cell esds are taken into account individually in the estimation of esds in distances, angles and torsion angles; correlations between esds in cell parameters are only used when they are defined by crystal symmetry. An approximate (isotropic) treatment of cell esds is used for estimating esds involving l.s. planes.

### Fractional atomic coordinates and isotropic or equivalent isotropic displacement parameters (Å^2^)

**Table:**

	*x*	*y*	*z*	*U*_iso_*/*U*_eq_	
F1	0.66861 (9)	0.65463 (11)	0.90225 (4)	0.0310 (2)	
F2	0.78633 (9)	0.79358 (9)	0.85702 (4)	0.02649 (19)	
F3	0.77343 (8)	0.56483 (9)	0.83505 (4)	0.02457 (18)	
F4	0.67911 (9)	0.90363 (10)	0.39138 (4)	0.02474 (19)	
F5	0.64920 (9)	1.02517 (9)	0.47416 (4)	0.02539 (19)	
F6	0.53035 (8)	0.83795 (9)	0.43956 (4)	0.02290 (18)	
O1	0.73476 (9)	0.72153 (11)	0.71987 (4)	0.0206 (2)	
O2	0.53551 (10)	0.81547 (11)	0.68469 (5)	0.0228 (2)	
H2	0.5616 (18)	0.826 (2)	0.6504 (8)	0.027*	
O3	0.50381 (10)	0.81283 (10)	0.80889 (5)	0.0220 (2)	
O4	0.61388 (9)	0.82629 (11)	0.57308 (4)	0.0203 (2)	
O5	0.82345 (10)	0.77145 (12)	0.61218 (4)	0.0223 (2)	
H5	0.7946 (17)	0.768 (2)	0.6476 (8)	0.027*	
O6	0.86887 (9)	0.86103 (11)	0.49365 (5)	0.02027 (19)	
C11	0.58366 (12)	0.70712 (14)	0.79025 (6)	0.0156 (2)	
C12	0.62748 (12)	0.75076 (14)	0.72772 (6)	0.0160 (2)	
C13	0.70474 (14)	0.67975 (15)	0.84652 (6)	0.0200 (3)	
C14	0.55428 (17)	0.95805 (16)	0.81927 (9)	0.0327 (4)	
H14A	0.48967	1.021062	0.831998	0.049*	
H14B	0.633739	0.957455	0.854131	0.049*	
H14C	0.573821	0.994618	0.778763	0.049*	
C15	0.50551 (12)	0.56621 (14)	0.77695 (6)	0.0162 (2)	
C16	0.53654 (13)	0.46020 (15)	0.73582 (6)	0.0201 (3)	
H16A	0.606416	0.475458	0.715198	0.024*	
C17	0.46551 (14)	0.33204 (15)	0.72487 (7)	0.0233 (3)	
H17A	0.487688	0.259446	0.697185	0.028*	
C18	0.36257 (14)	0.30976 (16)	0.75412 (7)	0.0242 (3)	
H18A	0.313193	0.222815	0.745973	0.029*	
C19	0.33183 (14)	0.41489 (16)	0.79538 (7)	0.0252 (3)	
H19A	0.261129	0.399788	0.815431	0.03*	
C21	0.75080 (12)	0.79338 (13)	0.49629 (6)	0.0151 (2)	
C22	0.72168 (12)	0.80054 (13)	0.56506 (6)	0.0156 (2)	
C23	0.65022 (13)	0.89092 (14)	0.45034 (6)	0.0179 (2)	
C24	0.98907 (14)	0.78807 (18)	0.52184 (7)	0.0269 (3)	
H24A	1.062021	0.848282	0.5162	0.04*	
H24B	0.995428	0.771765	0.5686	0.04*	
H24C	0.991539	0.694539	0.499883	0.04*	
C25	0.73698 (12)	0.63648 (13)	0.47147 (6)	0.0153 (2)	
C26	0.67573 (13)	0.53083 (14)	0.50129 (6)	0.0196 (3)	
H26A	0.644077	0.555168	0.538757	0.023*	
C27	0.66064 (14)	0.39003 (15)	0.47659 (7)	0.0222 (3)	
H27A	0.619756	0.318353	0.497469	0.027*	
C28	0.70527 (14)	0.35458 (15)	0.42156 (7)	0.0219 (3)	
H28A	0.694419	0.258779	0.404374	0.026*	
C29	0.76578 (15)	0.45913 (16)	0.39162 (7)	0.0247 (3)	
H29A	0.795872	0.434781	0.353697	0.03*	
C110	0.40383 (14)	0.54225 (15)	0.80757 (7)	0.0215 (3)	
H11A	0.383727	0.612817	0.836736	0.026*	
C210	0.78286 (14)	0.59938 (15)	0.41660 (6)	0.0214 (3)	
H21A	0.825871	0.669937	0.396217	0.026*	

### Atomic displacement parameters (Å^2^)

**Table:**

	*U*^11^	*U*^22^	*U*^33^	*U*^12^	*U*^13^	*U*^23^
F1	0.0344 (5)	0.0445 (6)	0.0150 (4)	−0.0067 (4)	0.0075 (3)	0.0032 (4)
F2	0.0266 (4)	0.0270 (4)	0.0238 (4)	−0.0092 (3)	0.0017 (3)	−0.0027 (3)
F3	0.0221 (4)	0.0232 (4)	0.0259 (4)	0.0044 (3)	0.0003 (3)	0.0043 (3)
F4	0.0308 (5)	0.0269 (4)	0.0178 (4)	0.0027 (3)	0.0083 (3)	0.0078 (3)
F5	0.0328 (5)	0.0140 (4)	0.0289 (4)	0.0045 (3)	0.0061 (3)	0.0008 (3)
F6	0.0177 (4)	0.0272 (4)	0.0223 (4)	0.0008 (3)	0.0016 (3)	0.0033 (3)
O1	0.0169 (4)	0.0271 (5)	0.0185 (4)	0.0023 (4)	0.0056 (3)	0.0041 (4)
O2	0.0204 (5)	0.0294 (5)	0.0193 (4)	0.0058 (4)	0.0057 (4)	0.0072 (4)
O3	0.0250 (5)	0.0146 (4)	0.0304 (5)	0.0009 (4)	0.0148 (4)	−0.0024 (4)
O4	0.0195 (5)	0.0246 (5)	0.0174 (4)	0.0030 (4)	0.0054 (3)	0.0005 (3)
O5	0.0193 (5)	0.0323 (5)	0.0151 (4)	0.0048 (4)	0.0036 (3)	0.0025 (4)
O6	0.0173 (4)	0.0193 (5)	0.0251 (5)	−0.0033 (4)	0.0065 (4)	0.0023 (4)
C11	0.0163 (6)	0.0154 (5)	0.0162 (5)	0.0012 (4)	0.0062 (4)	0.0000 (4)
C12	0.0168 (6)	0.0147 (5)	0.0167 (5)	−0.0004 (4)	0.0045 (4)	0.0005 (4)
C13	0.0230 (6)	0.0206 (6)	0.0165 (5)	−0.0035 (5)	0.0048 (5)	0.0000 (5)
C14	0.0373 (9)	0.0156 (7)	0.0504 (10)	−0.0018 (6)	0.0212 (7)	−0.0051 (6)
C15	0.0161 (6)	0.0148 (6)	0.0174 (5)	0.0006 (4)	0.0031 (4)	0.0019 (4)
C16	0.0195 (6)	0.0203 (6)	0.0213 (6)	−0.0008 (5)	0.0065 (5)	−0.0026 (5)
C17	0.0258 (7)	0.0191 (6)	0.0237 (6)	−0.0009 (5)	0.0031 (5)	−0.0043 (5)
C18	0.0239 (7)	0.0192 (6)	0.0269 (7)	−0.0045 (5)	0.0010 (5)	0.0025 (5)
C19	0.0242 (7)	0.0240 (7)	0.0295 (7)	−0.0037 (5)	0.0107 (5)	0.0045 (5)
C21	0.0154 (6)	0.0145 (5)	0.0160 (5)	−0.0006 (4)	0.0047 (4)	0.0011 (4)
C22	0.0189 (6)	0.0123 (5)	0.0156 (5)	−0.0012 (4)	0.0038 (4)	−0.0001 (4)
C23	0.0217 (6)	0.0155 (6)	0.0169 (5)	0.0003 (5)	0.0055 (5)	0.0019 (4)
C24	0.0164 (6)	0.0348 (8)	0.0296 (7)	−0.0006 (6)	0.0052 (5)	0.0022 (6)
C25	0.0157 (5)	0.0145 (5)	0.0160 (5)	0.0008 (4)	0.0039 (4)	0.0008 (4)
C26	0.0231 (6)	0.0170 (6)	0.0207 (6)	−0.0015 (5)	0.0094 (5)	−0.0002 (5)
C27	0.0251 (7)	0.0162 (6)	0.0267 (6)	−0.0037 (5)	0.0085 (5)	0.0002 (5)
C28	0.0235 (7)	0.0166 (6)	0.0245 (6)	0.0008 (5)	0.0033 (5)	−0.0035 (5)
C29	0.0333 (8)	0.0216 (7)	0.0216 (6)	0.0020 (6)	0.0110 (6)	−0.0031 (5)
C110	0.0235 (7)	0.0184 (6)	0.0250 (6)	0.0001 (5)	0.0109 (5)	0.0016 (5)
C210	0.0281 (7)	0.0187 (6)	0.0199 (6)	−0.0002 (5)	0.0108 (5)	0.0018 (5)

### Geometric parameters (Å, º)

**Table:**

F1—C13	1.3323 (15)	C16—H16A	0.95
F2—C13	1.3440 (16)	C17—C18	1.384 (2)
F3—C13	1.3370 (16)	C17—H17A	0.95
F4—C23	1.3467 (14)	C18—C19	1.387 (2)
F5—C23	1.3345 (15)	C18—H18A	0.95
F6—C23	1.3300 (16)	C19—C110	1.390 (2)
O1—C12	1.2152 (16)	C19—H19A	0.95
O2—C12	1.3095 (15)	C21—C25	1.5313 (17)
O2—H2	0.834 (14)	C21—C22	1.5456 (17)
O3—C11	1.4029 (15)	C21—C23	1.5489 (18)
O3—C14	1.4380 (18)	C24—H24A	0.98
O4—C22	1.2152 (16)	C24—H24B	0.98
O5—C22	1.3120 (15)	C24—H24C	0.98
O5—H5	0.866 (14)	C25—C210	1.3920 (17)
O6—C21	1.4096 (15)	C25—C26	1.3951 (17)
O6—C24	1.4407 (17)	C26—C27	1.3918 (19)
C11—C15	1.5298 (18)	C26—H26A	0.95
C11—C12	1.5430 (17)	C27—C28	1.3842 (19)
C11—C13	1.5511 (19)	C27—H27A	0.95
C14—H14A	0.98	C28—C29	1.385 (2)
C14—H14B	0.98	C28—H28A	0.95
C14—H14C	0.98	C29—C210	1.3899 (19)
C15—C16	1.3918 (18)	C29—H29A	0.95
C15—C110	1.3929 (18)	C110—H11A	0.95
C16—C17	1.3899 (19)	C210—H21A	0.95
			
C12—O2—H2	107.7 (13)	O6—C21—C22	112.79 (10)
C11—O3—C14	117.42 (11)	C25—C21—C22	109.51 (10)
C22—O5—H5	105.2 (12)	O6—C21—C23	102.04 (10)
C21—O6—C24	119.07 (10)	C25—C21—C23	109.69 (10)
O3—C11—C15	107.68 (10)	C22—C21—C23	107.49 (10)
O3—C11—C12	112.04 (10)	O4—C22—O5	124.79 (11)
C15—C11—C12	108.76 (10)	O4—C22—C21	122.24 (11)
O3—C11—C13	110.26 (10)	O5—C22—C21	112.93 (11)
C15—C11—C13	108.60 (10)	F6—C23—F5	108.29 (11)
C12—C11—C13	109.41 (10)	F6—C23—F4	106.58 (10)
O1—C12—O2	125.28 (11)	F5—C23—F4	106.68 (10)
O1—C12—C11	122.09 (11)	F6—C23—C21	112.71 (10)
O2—C12—C11	112.57 (11)	F5—C23—C21	111.56 (10)
F1—C13—F3	107.39 (11)	F4—C23—C21	110.71 (10)
F1—C13—F2	107.16 (10)	O6—C24—H24A	109.5
F3—C13—F2	106.91 (11)	O6—C24—H24B	109.5
F1—C13—C11	110.04 (11)	H24A—C24—H24B	109.5
F3—C13—C11	112.35 (10)	O6—C24—H24C	109.5
F2—C13—C11	112.70 (11)	H24A—C24—H24C	109.5
O3—C14—H14A	109.5	H24B—C24—H24C	109.5
O3—C14—H14B	109.5	C210—C25—C26	119.15 (12)
H14A—C14—H14B	109.5	C210—C25—C21	119.27 (11)
O3—C14—H14C	109.5	C26—C25—C21	121.55 (11)
H14A—C14—H14C	109.5	C27—C26—C25	120.52 (12)
H14B—C14—H14C	109.5	C27—C26—H26A	119.7
C16—C15—C110	119.55 (12)	C25—C26—H26A	119.7
C16—C15—C11	120.87 (11)	C28—C27—C26	119.91 (13)
C110—C15—C11	119.57 (11)	C28—C27—H27A	120.0
C17—C16—C15	120.14 (12)	C26—C27—H27A	120.0
C17—C16—H16A	119.9	C27—C28—C29	119.83 (13)
C15—C16—H16A	119.9	C27—C28—H28A	120.1
C18—C17—C16	120.26 (13)	C29—C28—H28A	120.1
C18—C17—H17A	119.9	C28—C29—C210	120.55 (12)
C16—C17—H17A	119.9	C28—C29—H29A	119.7
C17—C18—C19	119.71 (13)	C210—C29—H29A	119.7
C17—C18—H18A	120.1	C19—C110—C15	119.89 (13)
C19—C18—H18A	120.1	C19—C110—H11A	120.1
C18—C19—C110	120.41 (13)	C15—C110—H11A	120.1
C18—C19—H19A	119.8	C29—C210—C25	120.03 (12)
C110—C19—H19A	119.8	C29—C210—H21A	120.0
O6—C21—C25	114.82 (10)	C25—C210—H21A	120.0
			
C14—O3—C11—C15	−175.55 (12)	O6—C21—C22—O4	138.38 (12)
C14—O3—C11—C12	−55.99 (16)	C25—C21—C22—O4	−92.42 (14)
C14—O3—C11—C13	66.12 (15)	C23—C21—C22—O4	26.69 (16)
O3—C11—C12—O1	143.91 (12)	O6—C21—C22—O5	−43.73 (14)
C15—C11—C12—O1	−97.17 (14)	C25—C21—C22—O5	85.47 (13)
C13—C11—C12—O1	21.32 (17)	C23—C21—C22—O5	−155.42 (11)
O3—C11—C12—O2	−38.72 (15)	O6—C21—C23—F6	173.14 (10)
C15—C11—C12—O2	80.20 (13)	C25—C21—C23—F6	50.98 (13)
C13—C11—C12—O2	−161.31 (11)	C22—C21—C23—F6	−68.02 (13)
O3—C11—C13—F1	50.84 (14)	O6—C21—C23—F5	−64.78 (12)
C15—C11—C13—F1	−66.92 (13)	C25—C21—C23—F5	173.06 (10)
C12—C11—C13—F1	174.49 (10)	C22—C21—C23—F5	54.07 (13)
O3—C11—C13—F3	170.44 (10)	O6—C21—C23—F4	53.86 (12)
C15—C11—C13—F3	52.68 (13)	C25—C21—C23—F4	−68.29 (13)
C12—C11—C13—F3	−65.90 (13)	C22—C21—C23—F4	172.71 (10)
O3—C11—C13—F2	−68.70 (13)	O6—C21—C25—C210	−39.48 (16)
C15—C11—C13—F2	173.53 (10)	C22—C21—C25—C210	−167.56 (12)
C12—C11—C13—F2	54.95 (14)	C23—C21—C25—C210	74.70 (15)
O3—C11—C15—C16	156.05 (12)	O6—C21—C25—C26	142.61 (12)
C12—C11—C15—C16	34.43 (16)	C22—C21—C25—C26	14.53 (16)
C13—C11—C15—C16	−84.56 (14)	C23—C21—C25—C26	−103.21 (13)
O3—C11—C15—C110	−25.42 (16)	C210—C25—C26—C27	0.0 (2)
C12—C11—C15—C110	−147.04 (12)	C21—C25—C26—C27	177.94 (12)
C13—C11—C15—C110	93.96 (14)	C25—C26—C27—C28	−0.8 (2)
C110—C15—C16—C17	0.7 (2)	C26—C27—C28—C29	0.5 (2)
C11—C15—C16—C17	179.24 (12)	C27—C28—C29—C210	0.4 (2)
C15—C16—C17—C18	0.8 (2)	C18—C19—C110—C15	1.6 (2)
C16—C17—C18—C19	−1.1 (2)	C16—C15—C110—C19	−1.9 (2)
C17—C18—C19—C110	−0.1 (2)	C11—C15—C110—C19	179.57 (12)
C24—O6—C21—C25	−51.12 (15)	C28—C29—C210—C25	−1.1 (2)
C24—O6—C21—C22	75.29 (14)	C26—C25—C210—C29	0.9 (2)
C24—O6—C21—C23	−169.69 (11)	C21—C25—C210—C29	−177.05 (13)

### Hydrogen-bond geometry (Å, º)

**Table:**

*D*—H···*A*	*D*—H	H···*A*	*D*···*A*	*D*—H···*A*
O2—H2···O4	0.83 (1)	1.83 (1)	2.6616 (13)	173 (2)
O5—H5···O1	0.87 (1)	1.83 (1)	2.6801 (13)	169 (2)
C14—H14*B*···F2	0.98	2.2	2.840 (2)	122
C14—H14*C*···O2	0.98	2.53	3.080 (2)	115
C24—H24*B*···O5	0.98	2.22	2.8685 (18)	123
